# Impact of Met-Expectation of Athletic Justice on Athletic Satisfaction and Organizational Commitment via Leader–Member Exchange among Elite Saudi Arabian Athletes

**DOI:** 10.3390/bs13100836

**Published:** 2023-10-13

**Authors:** Seungmo Kim, Taeyeon Oh, Adam Love, Majed Essa Alahmad

**Affiliations:** 1 Department of Sport, Physical Education and Health, Hong Kong Baptist University, Kowloon Tong, Hong Kong; kimsm@hkbu.edu.hk; 2 Seoul AI School, aSSIST University, Seoul 03787, Republic of Korea; 3 Department of Kinesiology, Recreation and Sport Studies, University of Tennessee, Knoxville, TN 37996, USA; alove1@utk.edu; 4 Department of Sport and Recreation Management, College of Sport Sciences and Physical Activity, King Saud University, P.O. Box 145111, Riyadh 4545, Saudi Arabia; majalahmad@ksu.edu.sa

**Keywords:** perception of fairness, coaching behaviors, athletes’ attitudes, social exchange

## Abstract

Athletes’ perceptions of fairness toward coaching behaviors and decisions can play a crucial role in the development and maintenance of a strong coach–athlete relationship. However, scholars have given limited attention to athletes’ perceptions of fairness. Therefore, the current study aimed to explore the relationship between coaches and athletes by applying the concept of organizational justice based on met-expectation theory. The primary objective of the study was to empirically examine the direct and indirect relationships between the met-expectation of athletic justice and athletes’ attitudinal outcomes, such as athletic satisfaction and organizational commitment, through leader–member exchange (LMX). Data were collected from 289 elite athletes (238 men and 51 women) in the Kingdom of Saudi Arabia using a mixed-mode approach (paper-and-pencil and online surveys). The results of Structural Equation Modeling indicated that when athletes perceive that their expectations of fair treatment are met, it positively affects their satisfaction. This relationship is partially influenced by the quality of their relationship with their leader. However, the findings also suggest that while the athletes’ met-expectation of athletic justice has a positive effect on their satisfaction, it does not have a significant impact on their commitment to the team. The findings provide insight about important work-related outcomes by validating the coach–athlete relationship based on met-expectation of athletic justice. The findings can be utilized to improve athlete satisfaction and commitment, leading to positive team and individual outcomes.

## 1. Introduction

Building a strong and trusting relationship between athletes and coaches is an essential component of developing a successful team [1,2]. A team led by a coach who fails to provide adequate support, direction, and motivation to their players may struggle to function cohesively, resulting in poor performance. In contrast, a team with a coach who fosters a positive and supportive relationship with their players can inspire them to work harder, communicate more effectively, and achieve their objectives. Thus, the quality of the athlete–coach relationship is likely to have a profound impact on a team’s performance, either facilitating or hindering their success [3,4,5]. While there are several important factors (i.e., leadership, communication, and support) that contribute to a strong athlete–coach relationship, athletes’ perceptions of fairness with respect to coaching behaviors can act as a crucial factor in building and maintaining that relationship [6,7,8,9]. However, the topic of athletes’ perceptions of fairness has received limited attention in the literature. Therefore, the current study explored the relationship between coaches and athletes by applying the concept of organizational justice, which refers to members’ perceptions of fairness in an organization [10], to the context of sport. Based on the existing organizational justice literature, it was expected that athletes’ perceptions of coaches’ decisions and behaviors would play a crucial role in shaping athletes’ satisfaction, commitment, effort, willingness to help, and team unity, all of which impact individual and team performance [6,7,11].

### Met-Expectation

Two prominent concepts related to understanding employees’ satisfaction and commitment are met-expectation and the psychological contract. Met-expectation relates to the extent to which an individual employee’s expectations regarding their work environment and employment relationship (e.g., salary, benefits, and responsibilities; [12]) are met by the employer. Specifically, met-expectation refers to the “discrepancy between what a person encounters on [the] job in the way of positive and negative experiences and what he expected to encounter” [13] (p. 152). In contrast, psychological contract refers to the unwritten, informal and implicit expectations and obligations (e.g., job security, recognition, respect, and opportunity for development) based on a mutual agreement that arise from the employment relationship [14]. Although both met-expectation and the psychological contract are related to the fulfillment of expectations, which may influence job satisfaction in the workplace [15], the theories refer to different aspects of the employee–employer relationship. In the field of sport management, there have been a limited number of studies [15,16,17,18] that have adopted such concepts related to organization members’ expectations.

Unlike previous studies [6,7,8,9] that simply examined the relationships between the perception of fairness toward coaching behaviors and athletes’ attitudinal or behavioral outcomes, the current study employed a different approach by incorporating the concept of met-expectation as a way to measure athletes’ perceptions of the alignment between their expectations (e.g., their role, playing time, and training within their team) and their experiences regarding coaches’ behaviors. According to the met-expectation concept, when organization members’ expectations are met, they are likely to have a higher level of satisfaction and commitment. For example, Kim et al. [15] applied the concept of met-expectation to examine the relationships between coaches’ met-expectation of organizational justice and their job satisfaction and commitment in intercollegiate athletics in the United States. Therefore, this study provides evidence supporting the significance of athletes’ met-expectations in relation to their role, playing time, and training within a team, as well as the influence of coaches’ behaviors in these domains. By comprehending and addressing these factors, coaches can make equitable and met-expected decisions, thereby fostering athletes’ overall satisfaction and commitment.

In the context of athlete–coach interactions, when athletes join a team, they tend to develop expectations of their roles, playing time, and performance on the team and compare those expectations with their actual experiences. Met-expectation theory suggests that when athletes’ actual experiences on the team align with the expectations, they are more likely to feel satisfied and commit to their team, which is likely to help improve individual or team performance. However, when athletes perceive that their coaches are unfair or unjust in contrast with their expectations, they are more likely to have negative emotions (e.g., anger, frustration, and resentment), which may have a negative impact on performance [19].

## 2. Conceptual Framework

**Figure 1:**
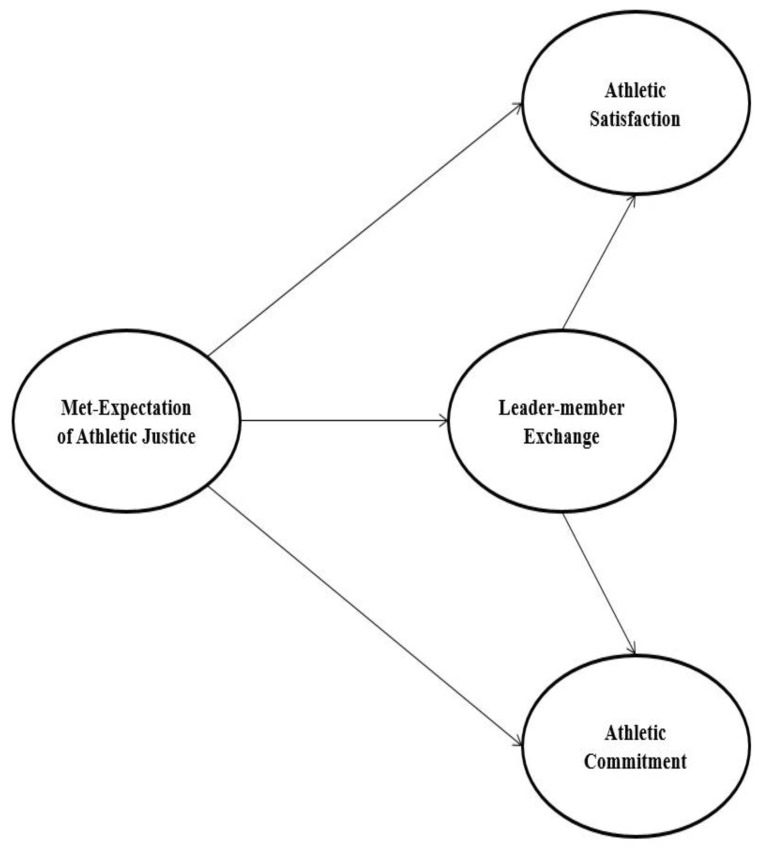
Conceptual framework.

The met-expectation theory provides a useful means of understanding how organization members’ perceptions of fairness in the workplace can impact their outcomes. In turn, a conceptual framework regarding the relationships between the met-expectation of athletic justice, leader–member exchange (LMX), and attitudinal outcomes (i.e., athletic satisfaction and team commitment) was developed, as shown in Figure 1. This framework describes how athletes’ expectations of fairness about coaching behaviors can impact their perceptions of the relationship with their coach (as measured by LMX), which in turn can affect their levels of satisfaction and commitment to their teams. Understanding these relationships yields valuable insights for coaches to improve their relationships with athletes and, ultimately, enhance team performance.

### 2.1. Organizational Justice in Sport

The concept of organizational justice has received considerable attention since the 1980s in the field of organizational behavior due to its significant impact on members’ attitudinal and behavioral outcomes [20]. Hums and Chelladurai [21] were the first to apply the concept of organizational justice to the context of sport when they examined stakeholders’ perceptions of fairness regarding resource distribution in U.S. intercollegiate athletics. Since this pioneering work, many researchers [8,22,23,24,25,26] have continued to adopt the concept to investigate perceptions of fairness among different stakeholders and its impacts on organization members’ attitudinal and behavioral outcomes in various settings, such as high school sports, university recreational departments, Olympic sports, and professional sport teams. Most studies on organizational justice in sports have focused on perceptions of fairness toward resource distribution (e.g., distribution of funds among sport teams in intercollegiate athletics) within sport and recreation organizations. However, the current study takes a novel approach by focusing on athletes’ perceptions of fairness toward coaches’ behaviors as target outcomes. In doing so, we term this type of organizational justice as “Athletic Justice” because coaches’ decisions in evaluating athletes’ abilities, assigning playing time, and allocating their roles and positions in the teams may serve a key role in influencing athletes’ perceptions of fairness within a team.

Organizational justice is a multi-dimensional construct [27]. According to the four-type model [28], employees in the workplace develop perceptions of fairness based on the presence or absence of fairness in four dimensions: (a) distributive justice (e.g., salary and promotion), (b) procedural justice (e.g., procedures or rules used to make outcome decisions), (c) interpersonal justice (e.g., quality of interpersonal treatment of the management in progress), and (d) informational justice (e.g., justifications and explanations provided regarding the determined outcomes). In turn, we conceptualize athletic justice as having the potential to be influenced by four aspects [29]: (a) distributive justice, where athletes perceive whether their coaches have been fair in evaluating their effort, ability, and contribution, allocating playing time, or assigning positions [30]; (b) procedural justice, where athletes perceive whether their coaches have used fair rules or processes in making outcome decisions [31]; (c) interpersonal justice, where athletes perceive whether coaches have been fair when communicating decisions, such as being respectful/disrespectful or sensitive/insensitive to athletes’ feelings [32]; and (d) informational justice, where athletes may perceive whether coaches have provided adequate justifications or explanations for their decisions [33]. Overall, the four dimensions of athletic justice, encompassing distributive, procedural, interpersonal, and informational justice, provide a comprehensive framework for understanding athletes’ perceptions of fairness within the team setting.

### 2.2. Outcomes of Organizational Justice

Based on the organizational justice literature, it is expected that there would be a positive relationship between the met-expectation of organizational justice and attitudinal or behavioral outcomes [15]. Athletes’ perceptions of fairness, based on their subjective evaluations regarding rules, policies, and procedures within their teams, can have a significant impact on their satisfaction [7,8,9,34]. When athletes perceive their athletic experiences as fair, they are more likely to report higher levels of satisfaction. Therefore, it is important for coaches to be aware that creating a fair and just athletic environment requires fair outcome distribution, decision-making processes, clear and transparent justifications for their decisions, and the respectful and sincere treatment of athletes, as such an environment can positively foster athlete satisfaction and commitment.

#### 2.2.1. Attitudinal Outcomes: Athlete Satisfaction and Team Commitment

Athlete satisfaction is defined as an athlete’s feeling of pleasure or happiness regarding their athletic experience [35]. It is considered to be an important factor that can impact athletes’ well-being and is linked to individual and team performance [35,36]. In fact, there is a substantial body of the literature that supports the positive role of athlete satisfaction on various outcomes, such as commitment and performance. Given the positive role of athlete satisfaction, personal factors (e.g., personality traits, self-esteem, and motivation) and environmental factors (e.g., coaching style, team cohesion, and organizational support) have been studied to understand how to develop a high level of athlete satisfaction. In the current study, we examined potential environmental factors, including met-expectations of organizational justice and leader–member exchange, that foster athlete satisfaction and ultimately lead to improved performance. Team commitment can be defined as the level of identification and attachment an athlete feels toward their team [37]. Numerous studies have found a positive relationship between employees’ perceptions of fairness and level of commitment to their organization [20], which suggests that when athletes feel they are being treated fairly within their team, they are more likely to feel committed to the team.

#### 2.2.2. Leader–Member Exchange as a Mediator

LMX, the quality of the relationship between a leader (e.g., coach) and their followers (e.g., athletes), can affect the attitudes of both the leaders and followers [38]. Coaches who demonstrate these qualities are more likely to develop high-quality LMX relationships with their athletes, leading to increased athlete satisfaction and team commitment [39]. Specifically, LMX involves, “(a) a system of components and their relationships, (b) involving both members of a dyad, (c) involving interdependent patterns of behavior, (d) sharing mutual outcome instrumentalities, and (e) producing conceptions of environments, cause maps, and values” [40] (p. 580).

The current study explored the impact of met-expectations, combined with organizational justice, on commitment. In other words, if athletes perceive that their expectations are being met in terms of fair treatment by their coach, they may be more likely to demonstrate commitment to the team. Numerous studies have found positive relationships between organizational justice and LMX [41] and between LMX and job satisfaction and commitment [42,43] and also a mediating effect of LMX between organizational justice and attitudinal outcomes [42] based on social exchange theory [44]. For example, athletes who have high-quality LMX relationships with their coaches are more likely to report higher levels of satisfaction with their sport experience and commitment to their team. Therefore, athletes’ perceptions of met-expectation are expected to positively influence LMX, which may have positive impacts on athletic satisfaction and commitment. Based on the existing literature, the following hypotheses were proposed regarding the constructs in the conceptual model.

**Hypothesis** **1:** *LMX will mediate the relationship between the met-expectation of fairness perception toward coaching behaviors and athlete satisfaction*.

**Hypothesis** **2:** *LMX will mediate the relationship between the met-expectation of fairness perception toward coaching behaviors and team commitment*.

## 3. Methods

### 3.1. Participants and Survey Procedure

The current study employed a cross-sectional research design. Data collection was undertaken through a mixed-mode approach combining traditional paper-and-pencil surveys with online surveys. Research Ethics Committee (REC) approval was obtained prior to data collection. Permission to collect data via offline or online surveys were acquired from each sport club before administering paper–pencil surveys or sending emails containing a survey link. The participants for this study were athletes based in the Kingdom of Saudi Arabia. A total of 356 responses were collected, out of which 289 were usable for the study, with the rest being excluded due to perceived insincerity or incomplete responses. The sample size aligns with the recommended criteria of Westland [45], who proposed a method for calculating the lower bound on sample size in CFA and SEM, which is expressed by Equation (1). Given the research design of the current study, comprising 22 observed variables and 4 latent variables, this calculation indicates that a sufficient sample size is 138. The final sample for the study comprised 238 men (82.3%) and 51 women (17.6%), with an average age of 27.5 years (SD = 9.05). Full demographic information, including the variety of sports played by the research participants, is provided in Table 1.

(1) $$n\geq 50r^{2}-450r+1100\;where\;r=\frac{number\;of\;observed\;variables}{number\;of\;latent\;variables}$$

### 3.2. Instrumentation

The present study consisted of four main variables: (a) met-expectation of fairness perception towards coaching behaviors, (b) LMX, (c) athlete satisfaction, and (d) team commitment. A set of questionnaires, comprising 35 items, was used to measure the data for these variables, as well as collect demographic information (e.g., age, gender, type of sport, and athletic career).

#### 3.2.1. Met-Expectation

The current study developed a scale of met-expectation of athletic justice based on the authors’ athletic justice scale [46]. While the original scale had 12 items across four dimensions (i.e., distributive, procedural, interpersonal, and informational justice), the current study adapted the scale to assess the met-expectation of athletic justice as a measure of overall justice. Sample items included “My coach addresses influence from administrators in a way I expect”, “My coach provides team members with feedback about decisions and their implementation in a way I expect”, and “My coach communicates their decisions in a way I expect”. Responses were recorded on a 7-point Likert scale ranging (1) ‘strongly disagree’ to (7) ‘strongly agree’.

#### 3.2.2. Outcome Variables

The remaining constructs—LMX, athlete satisfaction, and team commitment—were measured using existing scales in the field of organizational behavior. These variables were carefully chosen, building upon the existing literature, to ensure comprehensive and robust outcomes. The scales have been frequently employed in sport studies with contextual modifications. First, LMX for elite athletes was assessed using the 7-item scale developed by Scandura and Graen [47]. Sample items included “I always know how satisfied my coach is with what I do”, and “My coach certainly would be personally inclined to help solve problems”. Athletic satisfaction was assessed using a 5-item scale of job satisfaction by Judge et al. [48]. Sample items were “I feel satisfied with my team” and “I consider my team and my sport rather unpleasant. (Reversed coded)”. Finally, team commitment was measured by modifying the affective commitment component of Meyer and Allen’s [49] organizational commitment. Sample items include, “I would be very happy to spend the rest of my athletic career with my team” and “I do not feel like ‘part of the family’ in my team. (Reversed)”. In the context of sports, the reported reliabilities (α) of the scales were 0.94 for LMX [37], 0.82 for athlete satisfaction, and 0.82 for team commitment [36], respectively. All measurement items of the variables were adopted on a 7-point Likert scale, anchored from (1) ‘strongly disagree’ to (7) ‘strongly agree’.

### 3.3. Data Analysis

To ensure the robustness of our measurements, we conducted validation and reliability assessments using statistical tests like Confirmatory Factor Analysis (CFA). Subsequently, we utilized Structural Equation Modeling (SEM) to empirically test our hypotheses. First, the investigators performed Confirmatory Factor Analysis (CFA) on the collected data to verify the measurement validity and reliability of the variables. This statistical procedure confirmed the factor structure of our observed variables and the factor loads of each item. Items with factor loading values lower than 0.6 were removed, ensuring a high level of construct validity and reliability. In general, there are serval goodness-of-fit indices that CFA requires for validation of a measurement scale. According to Hair et al., [50], the chi-squared value (CMIN), Standardized Root Mean Residual (SRMR), Root-Mean-Square Error of Approximation (RMSEA), Comparative Fit Index (CFI) and Tucker–Lewis Index (TLI) indices are used to determine model fit, with CMIN requiring a *p*-value of 0.05 or less, SRMR < 0.08, RMSEA < 0.01, CFI > 0.9, and TLI > 0.9. After removing inappropriate items and ensuring model fit, the final questionnaire included 9 items for met-expectation, 7 items for LMX, 3 items for athlete satisfaction, and 3 items for team commitment, respectively. Following the CFA, investigators performed SEM to verify research hypotheses. This statistical procedure was chosen to uncover complex relationships between variables and to ascertain the strength and direction of these relationships. The SEM allowed for estimation of the direct, indirect, and total effects of the variables and testing of the proposed causal relationships between the met-expectation of athletic justice, LMX, athlete satisfaction, and team commitment.

## 4. Results

### 4.1. Measurement Validity and Reliability

The results of the study are organized into several sections that correspond to each stage of the data analysis. First, the CFA and associated model fit indices are reported, followed by tests of convergent and discriminant validity. Lastly, the Structural Equation Modeling (SEM) results are presented.

Table 2 presents descriptive statistics of the observed variables and the outcomes of the CFA. The model fit indices for the CFA demonstrated acceptable levels. Specifically, the chi-squared was x^2^ = 4594.612, degrees of freedom (df) were 231, CFI was 0.919, TLI was 0.908, RMSEA was 0.078, and the SRMR was 0.053, all of which were within the acceptable range, thus providing evidence for construct validity.

Next, we assessed the internal consistency of the constructs using Composite Reliability (CR). The CR values for met-expectation, athlete satisfaction, team commitment, and LMX were 0.924, 0.888, 0.865, and 0.890, respectively, exceeding the suggested threshold of 0.7 [44], which indicated the constructs have high internal consistency. We also considered the Average Variance Extracted (AVE), which were 0.578, 0.728, 0.683, and 0.688 for met-expectation, athlete satisfaction, team commitment, and LMX, respectively. As the AVE for each construct was above the recommended threshold of 0.5, this further affirmed the convergent validity of our measurement items [50]. The reliability of measurement items was verified using Cronbach’s alpha, with values of 0.923, 0.880, 0.865, and 0.915 for met-expectation, athlete satisfaction, team commitment, and LMX, respectively. All alpha values exceeded the commonly accepted threshold of 0.7, indicating that the measures were reliable [51]. Discriminant validity was ascertained through comparing the square root of the AVE of each construct with the correlation coefficients of each pair of variables, as shown in Table 3. The square root of the AVE for each variable was larger than the correlations between that variable and every other variable, which confirms discriminant validity [50].

### 4.2. Results of SEM

Turning to the SEM results, presented in Table 4, the model yielded an acceptable goodness-of-fit (x^2^ = 4594.621, df = 231, CFI = 0.919, TLI = 0.908, RMSEA = 0.078, SRMR = 0.053), which indicates the model fitted the data well (adopting the same criteria as CFA validation; [44]). Results from the SEMs revealed LMX partially mediated the relationship between met-expectation and athlete satisfaction, which supports hypothesis 1. The direct effect of ME on AS was estimated at 0.225, while the indirect effect via LMX was 0.234 (0.814 × 0.288). Thus, the total effect of met-expectation on athlete satisfaction was 0.459, suggesting LMX plays a significant mediating role. On the contrary, LMX was not found to mediate the relationship between met-expectation and team commitment, which does not support hypothesis 2. No significant direct or indirect effects were observed. This suggests that the impact of met-expectation of athletic justice on team commitment does not rely on the level of LMX. In sum, our data support the conceptual model and hypotheses, with the exception of the hypothesized mediating role of LMX in the relationship between the met-expectation of athletic justice and team commitment.

## 5. Discussion

The current study was designed based on prior research and theories in the field of organizational behavior to propose two research hypotheses. Specifically, we hypothesized that LMX would mediate the relationship between the met-expectation of fairness perception toward coaching behaviors and athlete satisfaction (H1) and the relationship between the met-expectation of fairness perception toward coaching behaviors and team commitment (H2). The results of the current study have important academic contributions and practical implications for the field of sport management and coaching.

First, the results of descriptive statistics indicate that most elite athletes in Saudi Arabia generally perceive their coaches as treating them fairly and with respect and develop quality relationship with their coaches. Additionally, the athletes report high levels of athletic satisfaction, which may reflect their positive experiences. However, they rated team commitment slightly lower than the other variables, which indicates an area for further consideration and improvement. Thus, coaches in this context should work on strategies that could enhance team commitment, such as team-building exercises and promoting a sense of shared goals and purposes among athletes. Given the high levels of athletic satisfaction and relatively strong commitment among athletes in Saudi Arabia, coaches have an opportunity to enhance overall team performance and cultivate a more cohesive and supportive athletic environment.

This study assessed athletes’ perception toward coaching styles and behaviors using the concept of organizational justice. Given the important influence of coaches on athletes’ experience and performance in teams, while numerous studies [52,53] have examined the relationship between coaching behaviors and athlete satisfaction, the current study provides a more nuanced perspective by examining the role of perceived fairness toward coaches’ decisions and behaviors on athletes’ attitudes. In addition, this study incorporated the concept of met-expectation in measuring athletic justice and expanded the understanding of athlete satisfaction and team commitment. The results of SEM analysis indicate that met-expectation regarding athletic justice had both direct and indirect impacts on athletic satisfaction, providing further evidence for the role of the met-expectation of athletic justice in the relationship between coaches and athletes. The overall results support a number of previous studies [9,15] by confirming that the congruence between justice perceptions and expectations positively impacts job satisfaction.

The current study also reinforces the importance of LMX and the quality of athlete–coach relationships on athletes’ attitudinal outcomes. The findings of the study supported a mediating role of LMX, showing that athletes’ satisfaction with their sports is directly linked to both the congruence between the athletes’ expectations and actual experiences regarding coaches’ decisions and behaviors in their teams, and their perceived quality of the social exchange relationships with their coaches. This highlights the importance of coaches’ fair and just behaviors and decisions in enhancing athlete satisfaction, which may ultimately lead to better overall team performance [6,7,8]. In this way, the current study contributes to the organizational behavior in the sport literature by empirically confirming the mediation effects of LMX in the relationship between met-expectation and outcome variables.

The findings of this study indicate that compared to athlete satisfaction, the met-expectation of athletic justice and LMX did not have a significant impact on team commitment. These findings suggest that while LMX plays a crucial role in promoting athlete satisfaction, it may not be as significant in promoting team commitment, which has implications for athlete retention and overall team performance. In the literature, LMX and POS are considered important social exchange variables to influence organizational commitment [46,54]. However, the findings of this study revealed the insignificance of LMX on team commitment in the context of sport. The descriptive statistics revealed that the mean score of team commitment was lower than the mean score of athletic satisfaction in the athletic context in Saudi Arabia, which may have contributed to the study’s insignificant findings regarding the roles of LMX and met-expectation on team commitment. These results also indicate other factors may be more important in promoting team commitment in sport. For example, athletes are often encouraged to adhere to a set of norms that stress the need to make sacrifices for the sake of the team [55,56]. In turn, athletes who are not satisfied may still have a high level of commitment to the team because of the expectation that they make sacrifices (including their own interests and satisfaction) for the betterment of the team. Overall, our study contributes to the growing body of research on organizational behavior in sports and provides a foundation for future research in this area.

The findings of the study provide several practical implications for coaches, athletes, and sport management professionals. First, this study reinforces the importance of coaches striving to create fair and just practices and team policies to enhance athlete satisfaction and performance. By understanding the importance of fair and just coaching practices in promoting athlete satisfaction, coaches can develop strategies to enhance athlete satisfaction and ultimately improve team performance. For athletes, this study emphasizes the importance of understanding the role of the met-expectation of athletic justice toward coaching behaviors in their satisfaction with their athletic experience. Athletes should be encouraged to communicate their expectations with their coach and provide feedback on their coaching experience. Additionally, athletes should be aware of the importance of their relationship with their coach in their overall satisfaction with their athletic experience. For sport management professionals, the study highlights the limitations of LMX in promoting team commitment. While LMX is an important factor in promoting athlete satisfaction, other factors, such as team cohesion, shared goals, and a sense of belonging, may be more important in promoting team commitment. Sport management professionals should consider these factors when developing strategies to promote team commitment and cohesion.

## 6. Limitations and Future Research

The current study contributes to our understanding of the effects of fair or unfair coaching behaviors on athletes’ attitudinal outcomes among elite athletes in Saudi Arabia. However, there are several limitations that need to be addressed in future research on the topic. First, this focus of this study was on elite athletes in Saudi Arabia. As such, readers should use appropriate caution when extrapolating the data to other populations, and future studies could be conducted in other cultural contexts or across different levels of sports (e.g., intercollegiate or interscholastic sports) to increase the generalizability of the findings in the athlete–coaching relationship. Second, this study relied on self-reported data from athletes, which may introduce biases and social desirability effects. For example, athletes have been hesitant to address negative attitudes toward their coaches. Future studies may utilize a mixed-methods approach to help further explore these issues. Observations or interviews could provide a more comprehensive understanding of the relationships between the met-expectation of athletic justice and outcomes. Furthermore, the study examined the effect of athletic justice on athletic satisfaction and team commitment through LMX. Future studies could incorporate other social exchange variables, such as perceived organizational support, and quality relationships between athletes and the team, to capture the full range of social exchange relationships between athletic justice and outcomes. Finally, a longitudinal design is recommended to examine the proposed relationships at multiple time points over an extended period to better understand the causal relationships between the met-expectation of athletic justice and attitudinal outcomes.

## 7. Conclusions

Coaches’ decisions greatly influence athletes’ satisfaction and commitment within the team. Therefore, this study advanced the knowledge of the relationships between coaches and athletes by examining athletes’ perceptions of fairness based on organizational justice and met-expectation theories. The results yielded insight into the direct and indirect relationships between the met-expectation of athletic justice and athletes’ attitudes through leader–member exchange based on social exchange theory. Specifically, leader–member exchange partially mediated the association between athletes’ met-expectation of athletic justice and satisfaction. However, no mediation effect was observed for team commitment. These results emphasize the importance of coaching fairness in improving athlete satisfaction. By validating the coach–athlete relationship based on met-expectation of athletic justice, teams could improve athlete satisfaction and commitment, benefiting both individuals and teams. Further research is needed to explore additional factors influencing the coach–athlete relationship and its consequences.

## Figures and Tables

**Table 1:** Demographic statistics of participants.

		*n*	Percent (%)
Gender	Male	238	82.3
	Female	51	17.6
Sports	Football	61	21.1
	Athletics	38	13.1
	Tennis	25	8.7
	Basketball	18	6.2
	Volleyball	15	5.2
	Cycling	14	4.8
	Taekwondo	14	4.8
	Wrestling	13	4.5
	Gymnastics	10	3.5
	Fencing	8	2.8
	Jujitsu	8	2.8
	Weightlifting	8	2.8
	Rowing	7	2.4
	Others	50	17.3

**Table 2:** CFA Results.

Construct	Item	Mean	S.D.	Loadings	CR	AVE	A
Met-Expectation					0.924	0.578	0.923
	My coach addresses influence from administrators in a way I expect.	4.88	1.89	0.747			
	My coach handles influence from political factors in a way I expect.	4.97	1.85	0.643			
	My coach addresses influence from team members’ parents in a way I expect.	5.16	1.66	0.739			
	My coach provides team members with feedback about decisions and their implementation in a way I expect.	4.93	1.98	0.688			
	My coach communicates their decisions in a way I expect.	4.76	1.97	0.785			
	My coach communicates with everyone on the team in a way I expect.	4.85	2.08	0.782			
	My coach meets my expectations by treating everyone on the team with respect.	5.72	1.69	0.795			
	My coach meets my expectations by treating everyone on the team in a polite manner.	5.57	1.77	0.829			
	My coach meets expectations by treating everyone on the team with dignity.	5.63	1.74	0.832			
Athlete Satisfaction					0.888	0.728	0.880
	Most days I am enthusiastic about my team and my sport.	6.08	1.44	0.759			
	I feel satisfied with my team.	5.56	1.67	0.912			
	I find real enjoyment in my team.	5.82	1.51	0.861			
Team Commitment					0.865	0.683	0.865
	I do not feel a strong sense of “belonging” to my team.	4.89	2.07	0.775			
	I do not feel “emotionally attached” to my team.	5.15	2.03	0.864			
	I do not feel like “part of the family” in my team.	5.18	2.11	0.839			
LMX					0.890	0.688	0.915
	I always know how satisfied my coach is with what I do.	5.23	1.87	0.728			
	My coach completely understands my problems and needs.	5.03	2.02	0.808			
	My coach fully recognizes my potential.	5.28	1.84	0.661			
	My coach certainly would be personally inclined to help solve problems	5.22	1.86	0.819			
	I certainly can count on my coach to “bail me out” at his/her expense when I really need it.	4.44	2.01	0.728			
	I have enough confidence in my coach that I certainly would defend and justify his/her decisions if he/she were not present to do so.	4.90	2.07	0.888			
	I would characterize my relationship with my coach as extremely effective.	5.22	1.84	0.814			
x^2^ = 4594.612, df = 231 *p* < 0.001, CFI = 0.919, TLI = 0.908, RMSEA = 0.078, SRMR = 0.053							

**Table 3:** Discriminant validity.

Variables	√AVE	ME	AS	AC	LMX
ME	0.760	1			
AS	0.853	0.459	1		
TC	0.826	0.182	0.389	1	
LMX	0.829	0.814	0.471	0.206	1

ME: met-expectation, AS: athlete satisfaction, TC: team commitment, LMX: leader–member exchange.

**Table 4:** Results of SEM.

To	From	Std. B	Std.err	*p*
LMX	ME	0.814 *	0.071	<0.001
AS	ME	0.225 *	0.087	0.046
	LMX	0.288 *	0.091	0.011
TC	ME	0.043	0.142	0.733
	LMX	0.171	0.148	0.174
x^2^ = 4594.621, df = 231 *p* < 0.001, CFI = 0.919, TLI = 0.908, RMSEA = 0.078, SRMR = 0.053				

ME: met-expectation, AS: athlete satisfaction, TC: team commitment, LMX: leader–member exchange. *: Significant at the 5% level.

## Data Availability

The data presented in this study are not publicly available to maintain confidentiality and privacy of the people who participated in the study.
